# Evaluation of an undergraduate occupational health program in Iran based on alumni perceptions: a structural equation model

**DOI:** 10.3352/jeehp.2017.14.16

**Published:** 2017-07-26

**Authors:** Semira Mehralizadeh, Alireza Dehdashti, Masoud Motalebi Kashani

**Affiliations:** 1 Faculty of Medicine, Iran University of Medical Sciences, Tehran, Iran; 2 Social Determinants of Health Research Centre, Department of Occupational Health, Semnan University of Medical Sciences, Semnan, Iran; 3 Department of Occupational Health, Kashan University of Medical Sciences, Kashan, Iran; Hallym University, Korea

**Keywords:** Education, Program evaluation, Occupational health

## Abstract

**Purpose:**

Evaluating educational programs can improve the quality of education. The present study evaluated the undergraduate occupational health program at the Semnan University of Medical Sciences in Semnan, Iran, with a focus on the associations between alumni perceptions of the learning environment and the outcomes of the occupational health program.

**Methods:**

A cross-sectional questionnaire survey was conducted among alumni of the undergraduate occupational health program. We asked alumni to rate their perceptions of the items using a 4-point Likert scale. The associations between alumni perceptions of the educational program and curriculum, faculty, institutional resources, and learning outcomes were modeled and described using structural equation modeling procedures.

**Results:**

A descriptive analysis of alumni perceptions indicated low evaluations for the administrative system, practical and research-based courses, and the number of faculty members. We found that a structural model of the evaluation variables of curriculum, faculty qualifications, and institutional resources significantly predicted undergraduate educational outcomes. The curriculum had direct and indirect effects on learning outcomes, mediated by faculty.

**Conclusion:**

The findings of our study highlight the usefulness of the structural equation modeling approach for examining links between variables related to the learning process and learning outcomes. Surveys of alumni can provide data for reassessing the learning environment in the light of the professional competencies needed for occupational health graduates.

## Introduction

In recent years, the academic literature and many national reports have recommended paying greater attention to the evaluation of educational programs [1]. The theoretical framework of our research was based on previous studies of evaluations in academic environments that suggested a range of variables important for understanding educational programs. Most evaluation studies in academic environments have focused on the curricula, teaching and learning methods, student-faculty interactions, and student outcomes [2,3]. In a previous study, researchers assessed the quality of faculty members according to achievements in education, training, research, and scholarship [4]. Previous research reported that faculty members who focused on improving the overall quality of their teaching could enhance the students’ level of motivation and higher-order thinking skills [5]. However, investigating whether faculty members influence overall academic performance and outcomes remains an important professional issue [6].

Previous studies have suggested that curriculum design may enhance students’ motivation for learning [7,8]. The factor of resources refers to the materials needed for teaching and training students. Learning resources such as the library, computer rooms, office space, and laboratory facilities should be assessed. Adequate resources must be available for a program to accomplish its mission [9]. While evaluation studies in higher education have proposed a range of variables for evaluating academic programs, few studies have investigated the relationships of variables such as curriculum, institutional resources, and properties of the faculty members with learning outcomes. Moreover, even less is known about alumni perceptions of educational programs in the context of evaluation [10].

**Fig. 1.**
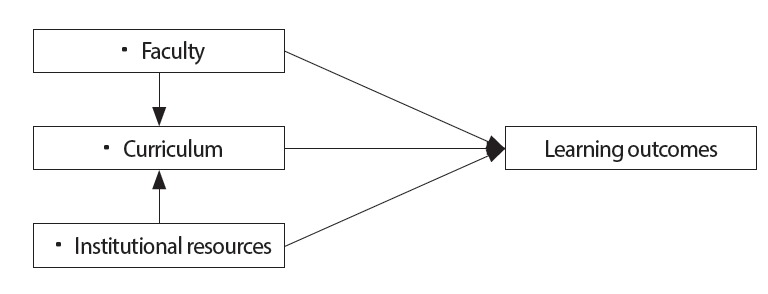
Hypothesized structural model of the evaluation study.

In Iran, the occupational health departments of medical sciences universities are responsible for the design and delivery of undergraduate occupational health educational and training programs. This study examined the associations between occupational health program alumni’s perceptions of the learning environment and their learning outcomes. We performed a comprehensive test of the associations among context, process, and outcome variables that were used for educational evaluation, based on responses from Iranian alumni engaged in occupational health activities. Fig. 1 presents the proposed model based on previous research background and theory.

## Methods

### Study design

A cross-sectional survey was performed from September 2015 to September 2016.

### Participants

The target subjects comprised alumni who graduated from a 4-year undergraduate occupational health degree program at the Semnan University of Medical Sciences between 1998 and 2014. The inclusion criteria were enrollment in the occupational health program at the Semnan University of Medical Sciences and professional engagement in occupational health activities. After obtaining the addresses of alumni from the educational affairs unit database, the questionnaires along with instructions were distributed to all alumni by mail and email.

### Instrument and data collection

Faculty members with experience in the occupational health program contributed to developing the alumni survey instrument. The study evaluated the perceived opinions of alumni on a variety of important topics to assess the undergraduate program in occupational health. The survey included descriptive items for alumni self-reporting on the quality of the undergraduate program, as well as items on the demographic characteristics of alumni, such as their involvement in postgraduate studies and employment status.

The first draft of our questionnaire consisted of 35 items distributed across 4 scales. That version was completed by 97 subjects to perform exploratory factor analysis. We deleted items with factor loadings less than 0.4, resulting in the elimination of 7 items. Of the study population, 126 alumni completed and returned the second version of the questionnaire. We performed confirmatory factor analysis to validate the structure of the survey instrument.

The final version of the questionnaire consisted of 28 items in 4 scales. The outcomes assessed the degree to which respondents perceived enhancements of their cognitive knowledge, skills, and abilities after their course of study. Five items assessed the program outcomes, with a focus on curriculum, learning sessions, and administration in the undergraduate occupational health program. Twelve items assessed the occupational health curriculum and educational program. The faculty construct measured the quality of faculty in terms of education and training. Six items assessed the faculty. The category of institutional resources described the equipment and facilities of the university, as measured by 5 items. Responses were given on a 4-point Likert frequency scale. Alumni perceptions of the undergraduate program were analyzed according to gender using multiple analysis of variance and comparison of means.

### Statistical analyses

A structural equation model was used to conduct confirmatory factor analysis and to evaluate the research model in the LISREL software program [11]. Our measurement model consisted of 4 latent constructs, including program outcomes; curriculum and program quality; and educational process, faculty, and resources as dependent variables and descriptive items as independent observed indicators.

Confirmatory factor analysis was carried out to confirm the validity of the instrument and the measurement model [11]. For all 4 latent variables, the estimation of measurement errors associated with each observed variable and indicator provided more accurate estimated parameters for inter-factor relationships. We estimated this to ensure that the chosen observed indicators defined the relevant latent construct accurately.

The Cronbach alpha coefficient was used to measure the internal consistency and reliability of our survey constructs. Table 1 presents the latent constructs, the measuring items, and their corresponding Cronbach alpha values. The acceptable level of reliability for every evaluation scale indicated the validity of the developed questionnaire when used among alumni in Iran.

The explanatory associations of the model were assessed by estimating the coefficient of determination of the endogenous dependent learning outcomes construct. In order to assess the extent to which the individual constructs were discriminated from each other by the survey instrument, we estimated the square root of average variance extracted for each construct and inter-construct correlations [11].

In our conceptual model, the curriculum and program, resources, and faculty variables were assumed to affect program outcomes. We estimated the goodness of fit of the proposed model by the chi-square test, goodness-of-fit index, root mean square error of approximation, and standardized root mean square residual, as suggested by Kline [11].

### Ethical approval

Ethical approval was obtained from the Ethics Committee Review Board of the research center of Semnan University of Medical Sciences (Reference code: IR.SEMUMS.REC.1395.233). We considered completed and returned questionnaires to indicate the provision of informed consent.

## Results

### Response rate

Data from a total of 126 alumni who answered and completed the survey were included in the study, resulting in an approximate response rate of 37%. Raw data were available from Supplement 1.

### Alumni perceptions of their educational program

Our survey measured alumni perceptions of the level of cognitive and behavioral knowledge they achieved by participating in the undergraduate occupational health program. Forty-eight percent of the participants agreed or strongly agreed with the item assessing learning outcomes. The item relating to their understanding of occupational health concepts received the highest score. Furthermore, alumni evaluated the overall structure of the curriculum and program as relatively appropriate. Seventy-six percent of the alumni reported strong agreement regarding clear announcements of the goals of the program, curriculum content and its availability, and educational materials.

However, they identified several areas requiring modifications and improvements. For the items related to administration system, laboratory courses, research and project internships, and the number of faculty members specialized in occupational health, over 40% of the responses indicated disagreement or strong disagreement. The majority of alumni reported agreement or strong agreement for the item on library facilities. However, the respondents presented poor evaluations of other items describing laboratory equipment and the number of technical staff.

Fifty-four percent of the alumni reported encouraging opinions on the items evaluating whether faculty members dedicated enough time to student instruction and had appropriate qualifications and experience in teaching and research, except for the items “faculty workload allowed them to fully carry out their role” and “faculty members provided students with various learning methods,” which received responses of disagree or strongly disagree by 43% of the alumni.

Our results indicate that for all educational program evaluation constructs, the variance in participants’ scores was on average moderate. Learning outcomes had the largest variance of all 5 constructs. The least variance was found for the educational curriculum and program.

Male alumni evaluated learning outcomes more favorably than female alumni (P< 0.05). However, female perceptions were more favorable toward faculty members and the curriculum (P< 0.05).

### Questionnaire validity

Table 2 shows the matrix of correlation residuals between the studied constructs. The results of our analysis confirmed the discriminant validity, because for each construct the square root of average variance was estimated to be higher than the correlations between constructs. This suggests that the survey instrument consisted of latent variables that were distinguished from each other.

### Structural equation model

**Fig. 2.**
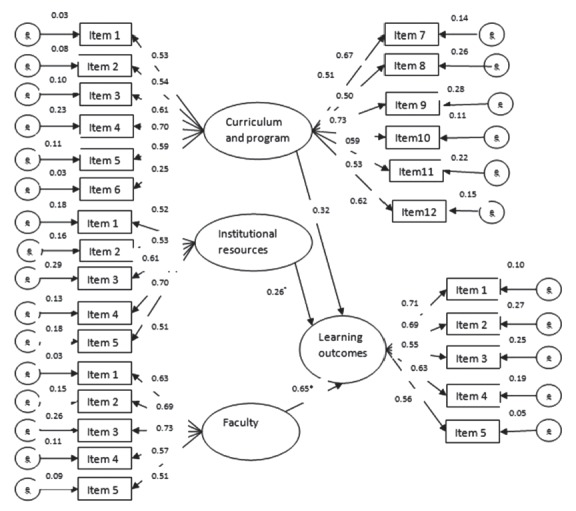
Structural equation modeling reflects the fact that curriculum and program, institutional structure, and faculty are related to program learning outcomes of the undergraduate occupational health program. The figures between latent variables indicate the standardized path coefficients for the relationships in the model (*P<0.05). The data were collected from a questionnaire survey administered to alumni from Semnan University of Medical Sciences.

The associations of alumni perceptions of the curriculum and program, faculty, resources, and learning experiences with the outcome variables were analyzed by means of structural equation modeling. The fit indices of the hypothesized initial and final models showed a relatively adequate fit to the data (Table 3), implying a non-significant difference between the model and the data. The standardized regression path coefficients presented in Table 4 indicate the extent to which each independent variable assessing perceptions of the curriculum, faculty, and resources was related to the dependent variable of learning outcomes. Statistically non-significant relationships were excluded from the hypothesized model to propose the final model. The final structural model is illustrated in Fig. 2.

Our analysis found that faculty perceptions had the largest positive effect on learning outcomes (β= 0.65). Therefore, the more positively alumni perceived the level of quality and activities of the faculty members, the more positively they perceived their learning achievements.

Perceptions of the curriculum were associated significantly and positively with learning outcomes. However, the curriculum had a direct effect of 0.32 and an indirect effect of 0.13 through the faculty variable, and thus a total effect of 0.51 on alumni learning outcomes. This means that the more positively alumni perceived the curriculum and program, the more they were satisfied with their learning experiences and the outcomes of their undergraduate program.

Finally, the institutional resources variable had a direct effect of 0.26 and an indirect effect of 0.11 on learning achievements through the curriculum and program. Therefore, the total effect of institutional resources on the learning outcomes was considerable. This implies that as alumni perceive and evaluate institutional resources more positively, they are likely to have a favorable evaluation of their learning outcomes.

The measurement of variance associated with the dependent learning outcomes variable was used to assess the explanatory influence of the proposed model. The coefficient of determination showed that 72% of the variation in the alumni perception of learning outcomes could be explained by their perceptions of the curriculum and program, faculty, and resources.

## Discussion

The purpose of this study was to present and analyze how alumni evaluated an undergraduate program in occupational health. We applied an experimental methodology capable of analyzing the degree of influence of curriculum and program, faculty performance, and institutional resources on program outcomes.

The current study found that faculty had a direct influence on the learning experience and outcomes, with the strongest association in the total structural model (0.65). This influence was around twice as strong as the influence of the curriculum and administrative procedures. This implies that faculty quality is important for explaining alumni perceptions of the undergraduate educational process and learning outcomes. In other words, experiencing more mentor support in the academic environment is highly likely to improve alumni perceptions of learning outcomes. This is consistent with previous research reporting that mentorship could enhance the learning process [6].

Our study revealed that curriculum and program administration both directly and indirectly influenced the outcomes variable through the faculty variable. A previous study recommended that evaluations of academic programs should be focused not only on curricula, but also on the activity of the faculty [5]. Research has strongly pointed out that student-faculty interactions are crucial to learners’ academic performance, outcomes, and satisfaction [11,12].

The present study clearly showed that faculty can play a crucial role in predicting educational outcomes. Therefore, academic institutions and departments should be careful in recruiting and evaluating faculty members. Academic departments offering an occupational health program at the undergraduate level should encourage faculty members to consider their interactions with learners, qualifications, expertise, and effective learning methods. In academic settings, quality enhancement practices may be introduced to improve the skills of faculty members. Meanwhile, previous reports have emphasized that departments must provide support for faculty members to incorporate research and project-based learning into their teaching approach. This will assist students to learn how to deal with real situations that they may encounter in their future careers [7,11].

The quality of the curriculum can be considered a decisive factor in improving learning outcomes. Alumni who evaluated the content of the curriculum as suitable more often reported improvements in their learning ability, teamwork, and field knowledge. An earlier study indicated that curricular design had a positive effect on the judgements of graduates concerning the usefulness of the educational program for their current jobs [11]. The type of instructional method in higher education plays an important role in providing students with knowledge, skills, and attitude-based competencies in their specific professional environment [7,11]. An educational approach that engages students in problem-solving, projects, and research in which learners structure and organize their knowledge, simulating situations that they may face in the real work environment, may be helpful for improving educational outcomes and preparing graduates for their future careers [5,12].

In our study, alumni believed that little attention had been paid to incorporating research and projects in the undergraduate occupational health program. We suggest that occupational health departments integrate problem-solving and project-based learning into the curriculum and incorporate practice-based research into their professional educational program. However, to achieve this successfully, faculty members interested in engaging in research activities should be recruited [13].

In conclusion, the network of associations in the proposed structural model revealed that learning outcomes were statistically related to the curriculum and program, institutional resources, and faculty. The structural equation model is valuable for program evaluation and obtaining information from alumni, because it allowed us to obtain useful data for internal program planning and for assessing the fit of the educational environment and occupational health discipline curriculum with alumni’s professional opportunities. However, a limitation of the present study is that we analyzed data from a single academic institution with a focus on occupational health education, and the variables described might be peculiar to this area of study. It should be emphasized that because the data were collected through a cross-sectional design, our analysis was limited to identifying associations between measured latent variables, rather than causality.

## Figures and Tables

**Table 1.** Survey instrument containing items used to measure each latent construct and the relevant Cronbach alpha

Constructs	Evaluation items	Cronbach alpha
Alumni learning outcomes	1. Have sufficient knowledge in occupational health and be able to understand occupational health concepts.	0.73
	2. Have the ability to undertake a risk assessment and provide solutions to occupational health problems.	
	3. Have the ability to use sampling and analytical techniques and the skills required in occupational health engineering practice.	
	4. Have the ability to carry out a project as part of a team.	
	5. Understand the needs of self-based learning.	
Curriculum and program	1.The goals of undergraduate occupational health program were clearly communicated to students.	0.71
	2. The curriculum included courses related to the occupational health program.	
	3. The educational and training environments were supportive for learners.	
	4. Sufficient educational materials and information were available.	
	5. The institution's administration system supported students in carrying out research projects.	
	6. There were enough faculty members to meet the needs of the undergraduate occupational health program.	
	7. Course details were available in written form to students.	
	8. Course sessions were well organized.	
	9. Laboratory courses facilitated the learning process.	
	10. The program enhanced interaction among students and between students and faculty.	
	11. The research/project internship in a work environment setting facilitated professional practice.	
	12. When I completed the program, I felt more competent regarding occupational health issues.	
Institutional resources	1. The library had useful resources, database, and search engines.	0.63
	2. Computer and information technology facilities.	
	3. Laboratory equipment for supporting learning and research.	
	4. Technical staff to support students.	
	5. Space in university buildings for student activities.	
Faculty members	1. Faculty members dedicated sufficient time to instruct students and support the learning process.	0.71
	2.The faculty had appropriate qualifications and expertise in the occupational health area, suitable for student learning.	
	3. Faculty workload allowed them to fully carry out their role.	
	4. Faculty members provided students with various learning methods.	
	5. Faculty members had teaching and research experiences appropriate for occupational health education.	

**Table 2.** Correlations between constructs and the roots of average variance

Construct	Learning outcomes	Undergraduate education curriculum and program	Institutional resources	Faculty members
Learning outcomes	0.45^a)^			
Undergraduate education curriculum and program	0.23^*^	0.62^a)^		
Institutional resources	0.14^*^	0.19^*^	0.55^a)^	
Faculty members	0.29^*^	0.21^*^	0.16^*^	0.71^a)^

* P<0.05.

a) Square roots of average variance.

**Table 3.** Results of fit indices for the hypothesized and final models

	Measures of fit					
	Degrees of freedom	Chi-square	P-value	Goodness-of-fit index	Root mean square error of approximation	Standardized root mean square residual
Hypothesized model	730	1415.3	0.68	0.87	0.02	0.07
Final model	716	1130.87	0.65	0.91	0.05	0.07

**Table 4.** Standardized regression coefficients and t-values for the associations of latent constructs in the final structural model

Association	Path coefficient estimate	t-value
Curriculum and program → learning outcomes	0.32	4.02^*^
Institutional resources → learning outcomes	0.26	5.20^*^
Institutional resources → curriculum and program	0.11	0.43
Faculty → learning outcomes	0.65	5.34^**^
Faculty → curriculum and program	0.25	1.65

* P<0.005,

** P<0.001.

## Supplementary materials

Supplement 1. Data file is available from https://dataverse.harvard.edu/dataset.xhtml?persistentId=doi:10.7910/DVN/F1RSQA

Supplement 2. Audio recording of the abstract.

## References

[1] Bredtmann J, Crede CJ, Otten S (2013). Methods for evaluating educational programs: does Writing Center participation affect student achievement?. Eval Program Plann.

[2] European Agency for Safety and Health at Work (2010). Mainstreaming occupational safety and health into university education.

[3] Lewallen LP (2015). Practical strategies for nursing education program evaluation. J Prof Nurs.

[4] Arimoto A, Gregg MF, Nagata S, Miki Y, Murashima S (2012). Evaluation of doctoral nursing programs in Japan by faculty members and their educational and research activities. Nurse Educ Today.

[5] Dehdashti A, Mehralizadeh S, Kashani MM (2013). Incorporation of project- based learning into an occupational health course. J Occup Health.

[6] Brown T, Williams B, McKenna L, Palermo C, McCall L, Roller L, Hewitt L, Molloy L, Baird M, Aldabah L (2011). Practice education learning environments: the mismatch between perceived and preferred expectations of undergraduate health science students. Nurse Educ Today.

[7] Watson MK, Lozano R, Noyes C, Rodgers M (2013). Assessing curricula contribution to sustainability more holistically: experiences from the integration of curricula assessment and students’ perceptions at the Georgia Institute of Technology. J Clean Product.

[8] Lidice A, Saglam G (2013). Using students’ evaluations to measure educational quality. Procedia Soc Behav Sci.

[9] Cobb KA, Brown GA, Hammond RH, Mossop LH (2015). Alumni-based evaluation of a novel veterinary curriculum: are Nottingham graduates prepared for clinical practice?. Vet Rec Open.

[10] YuekMing H, Manaf LA (2014). Assessing learning outcomes through students’ reflective thinking. Procedia Soc Behav Sci.

[11] Kline RB (2011). Principles and practice of structural equation modeling.

[12] Victor G, Ishtiaq M, Parveen S (2016). Nursing students’ perceptions of their educational environment in the bachelor’s programs of the Shifa College of Nursing, Pakistan. J Educ Eval Health Prof.

[13] Reinhold K, Siirak V, Tint P (2014). The development of higher education in occupational health and safety in Estonia and selected EU countries. Procedia Soc Behav Sci.

